# Applying Community-Engaged Pedagogy to Culinary Medicine: Why and How Community Nutrition Enriches Culinary Medicine Curricula

**DOI:** 10.1177/15598276251371573

**Published:** 2025-08-26

**Authors:** Ariela Zebede, Lev Krasnovsky, Jarrett Stein, Cory Bowman, Horace M. DeLisser

**Affiliations:** 6572 Perelman School of Medicine at the University of Pennsylvania, Philadelphia, PA, USA (AZ, LK, HMD); Netter Center for Community Partnerships, 6572 University of Pennsylvania, Philadelphia, PA, USA (JS, CB)

**Keywords:** culinary medicine, community engagement, community-engaged pedagogy, community-engaged learning, medical education, nutrition, social determinants of health, interprofessional collaboration, cultural competency, community-based learning

## Abstract

Diet is a critical determinant of health, yet nutrition education remains underemphasized in medical school curricula, leaving physicians inadequately prepared to discuss nutrition with patients. Culinary Medicine (CM), a growing field blending nutritional science with practical culinary skills, offers a promising model for enhancing medical trainee competence in dietary counseling. However, many CM programs focus primarily on nutrition and culinary training, often underutilizing the educational potential of community engagement. This article advocates for the integration of community-engaged pedagogy (CEP) as a vital third pillar in CM curricula. CEP fosters reciprocal learning between medical trainees and community members, which deepens student understanding of patients’ lived experiences with social determinants of health, enhances the practicality of dietary recommendations, and promotes novel forms of interprofessional collaboration. The authors provide recommendations and examples of how CEP can be embedded into CM training and call for rigorous evaluation of CEP integration to ensure that outcomes align with the goals of both students and community partners. Ultimately, as CM courses grow in popularity and become incorporated into more academic curricula, it is important to ensure that CEP becomes established as a core element of CM programming.

“CEP in CM presents a clear opportunity for students to take what they have learned further: from the classroom, to the kitchen, to the community.”

## Introduction

The significant impact of diet on health cannot be overstated, with a large body of data demonstrating that a poor diet is responsible for more deaths globally than any other risk factor.^1^ Beyond the well-known diet-related diseases of hypertension, diabetes, and cardiovascular disease, diet quality has also been shown to play a role in the development of a number of other conditions including cancers, neurodegenerative diseases, and mental illness.^2^ Despite the critical influence of diet on health, nutrition education has yet to acquire a commonplace, rigorous position in medical school training, leading to physicians who are inadequately prepared to discuss nutrition with patients.^3-5^

An emerging curricular response to address nutrition training deficits in medical school education has been the pedagogical approach of Culinary Medicine (CM). Defined as an “evidence-based field in medicine that blends the art of food and cooking with the science of medicine,”^6^ CM typically involves nutrition education combined with culinary didactics and has been shown to help students translate nutritional concepts and recommendations into practical skills and patient advice.^7-9^ Nutrition education and culinary training are often regarded as the core pillars of CM, but we believe that a third pillar, community engagement, merits equal recognition. In this *Perspectives* piece, we propose that embedding community engagement into CM training provides opportunities to strengthen practical applications of student learning, benefit community partners, and address important areas of focus in the teaching of social medicine.

## Why Community Engagement is Important to Culinary Medicine

Currently, relatively few CM programs incorporate community-based components into their training. A 2022 review of 24 published programs found that only 9 included any type of engagement with community members.^8^ Most of these existing initiatives center on delivering cooking classes or nutritional instruction, typically positioning institutional partners as “teachers” and community members as “learners.” At the University of Chicago’s Culinary Medicine program, for example, medical students co-teach culinary medicine classes with faculty and chefs to community members.^10^ The program has demonstrated significant improvement in medical students’ counseling skills and in community members’ understanding of chronic disease and kitchen skills.

Despite the clear value of these community-based initiatives, they may inadvertently reinforce hierarchical dynamics that limit the potential for medical trainees to view community members as sources of knowledge.^11^ We believe there remains untapped potential in reimagining these efforts not solely as opportunities to teach, but as platforms for reciprocal learning—where community members are also regarded as educators and co-creators of knowledge. For several reasons, the under-utilization of truly collaborative, community-engaged learning in CM programming represents a missed opportunity to foster cultural and structural competence through the lens of nutrition and lifestyle training.

First, for medical trainees to apply nutrition and lifestyle concepts in clinical settings, they must understand the lived experiences of their patients and the sociocultural factors that impact their lives. This has been demonstrated repeatedly in the literature, with cultural inclusion in counseling being associated with “higher rates of adherence to dietary changes and improvements in disease management.”^12^ While the teaching of social medicine is an established feature of medical school curricula, there are limitations to the depths of self-reflection and the extent of transformation in thinking that can be achieved with classroom-based pedagogy alone.^13^ In contrast, there is transformative learning that arises when students venture outside of their familiar classroom spaces, engaging with their putative patients outside of the clinical setting and seeing hierarchies flattened and power dynamics reversed. As it stands, multiple studies already demonstrate a growing desire among medical trainees to let patient lived experiences speak for themselves—a finding that CM curricula are well suited to respond to.^14-18^

Second, nutrition knowledge and culinary experience alone do not translate into improved patient health outcomes; these concepts must be communicated to and ultimately implemented by patients to make a difference. Meaningful community engagement can help trainees identify when standard recommendations are infeasible, unfamiliar, or simply overwhelming for the patient before them. With this perspective, trainees can learn to provide context-driven recommendations that are guideline-informed but tailored to specific circumstances. For example, a trainee might learn that advising a patient to incorporate more fresh vegetables into their diet is impractical for a patient with no nearby grocery stores or a reliable refrigerator to store fresh produce. Instead, they may recommend affordable canned options with low sodium content. Ultimately, practical recommendations that are implemented are more valuable than idealized recommendations which are not.

Lastly, community-engaged CM provides an opportunity to teach interprofessionalism in a novel way. Typical CM programs have included interprofessional collaboration with registered dietitians, nutrition scientists and public health experts. Community-engaged CM, however, includes community partners as additional colleagues for addressing social determinants of health. Such behavior fosters sustainability of the relationship and ensures that programming benefits all parties equitably. Shared involvement in problem identification and solution development also promotes empowerment and more durable project outputs.^19^ This helps create a platform from which communities can more strongly articulate their needs, priorities, and challenges in a manner that retains agency.

## Community-Engaged Pedagogy

We propose community-engaged pedagogy (CEP) as the guiding framework for integration of impactful community experiences into CM curricula. Also known as community-engaged learning, CEP emphasizes reciprocal learning in real-world settings, where students and community members co-learn.^14,20-22^ This approach fosters experiential learning, and problem-oriented (versus content-oriented) discussion that better aligns with the educational preferences of adult learners.^23^ Simultaneously, it breaks down traditional hierarchical structures between academic institutions and community members.

Emphasis on bidirectional learning and shared agency in decision-making distinguishes community-engaged pedagogy from service-learning.^20^ In traditional service-learning, there is an explicit connection between the educational plan and the performance of community service, but no necessary focus on ensuring that involved parties benefit from the relationship to similar extents.^20^ In offering this critique, we do not discount the significant learning that can be attained through these established approaches. However, this type of pedagogy does risk succumbing to a “safari-like” curriculum, with students acting as “tourists” among the disadvantaged.^24,25^ CEP, in contrast, can be thought of as a new generation of service-learning that emphasizes reciprocity, agency, and collaboration.^26^

While both service-learning and CEP can contextualize social determinants of health (SDOH) for medical students, simply understanding SDOH is not the aim of CEP. The aim instead is to meaningfully engage with the realities of social disparities and to address these challenges as partners with community members. From a practical standpoint, CEP enables educators to teach SDOH in the concrete, rather than the abstract.^24^ In fact, CEP can address the broader definition of SDOH, presented by Stonington and Holmes, which includes not only broad socioeconomic inequalities, but also more subtle “forces such as cross-cultural miscommunication and power differentials in provider–client interactions.”^24,27^ Ideally, this broader approach not only provides students tangible skills, but also inspires motivation to practice in community settings. CEP supports curricular integration, advances community health education, and empowers students as collaborators in addressing local needs.^28-30^

## Integration of CEP into CM Programming

CEP in culinary medicine courses and programming can take many forms. A universal approach is impractical due to the uniqueness of every institution-community relationship, but some examples, either possible or currently in practice, may include: co-organized dinner events by students and community members, co-developed and co-led nutritional workshops for youth development programs, shared learning experiences on combined field trips to farms and community gardens, collaborative advocacy efforts around food access, joint entrepreneurship ventures, and joint consulting services for local food businesses.^10,28,31-36^

Regardless of form, we posit several key essentials based on the literature and our own experiences to integrate robust community experience into medical student culinary medicine training. First, meaningful community partnership should include long-term commitments to collaboration. One-off community-based initiatives, as exist in many current CM courses,^8,9^ are unlikely to sustain the relationships necessary for program participants to have mutually transformative experiences. In this regard, it may be preferable to collaborate on ongoing community-focused efforts, rather than independent initiatives confined to discrete academic cycles. Importantly, for durability, what is developed needs to be integrated as a core element into the established CM programming and not adopted merely as an add-on to existing instruction. A mutually participatory approach to design and implementation should guide the process. To that end, evaluation of CEP implementation efforts should assess both institutional education metrics and community impact priorities. Importantly, publications on CEP initiatives should include, as appropriate, community members as co-authors.

As an example of the integration of CEP into a CM course, we highlight our experience with a pre-clerkship CM elective at the Perelman School of Medicine at the University of Pennsylvania. In this yearlong elective, medical students in the course collaborate with local high school students to develop the necessary skills to run community dinners, an ongoing initiative in the partner high school to promote fellowship, wellness, and youth leadership through food. During the spring semester of year one, medical students attend high school student-led dinners as assistants to learn about the relationships community members have with food and their food environments. In the fall of year two, high schoolers attend medical school culinary labs to workshop recipes for upcoming community dinners and learn about nutrition science. A combined field trip to a local regenerative farm further extends the shared learning. Joint reflections drive future menu development, and contact over two academic semesters helps promote partnership longevity. Curricular assignments, lectures, and recipes are derived from this arrangement, emphasizing its critical place in the course. Our model of CEP-CM would not have been possible without institutional resources, external grant support, and a partnership with a trusted, community-focused university center that helped connect our program with local partners.

As a further example of CEP-CM integration, we note an elective for medical students at the Penn State College of Medicine that brings together fourth year students and senior citizens for an intergenerational learning experience.^31,32^ Conducted in a local older adult center, the students and older adults collaboratively prepare meals, discuss the sociocultural and relational aspects of food, and mutually learn from each other, with the older adults sharing their personal challenges around food and providing practical insights to the students. In presenting these examples of CEP-CM integration, we acknowledge that developing these efforts in elective formats limits their reach to the broader student body but is currently what is feasible given the realities of limited funding and curricular time in medical education. That said, elective experiences do provide opportunities to collaborate with other courses (elective or otherwise) to grow CEP integration more broadly.

To build CEP programs effectively, institutions need a broad range of invested team members: faculty or clinicians with expertise in nutrition and community engagement (whether they be physicians, registered dietitians, nutrition scientists, or public health professionals), community leaders with deep roots in the community, chefs and others with culinary expertise in healthy cooking and student advocates with a passion for preventive nutrition and lifestyle medicine who can influence their peers and engage school administration. Given the inevitable turnover in the members of this type of team, being intentional about recruitment and refreshment of team members and succession planning is essential for the sustainability of these initiatives. Although we recommend CEP as a best practice, we recognize that it may not be possible to fully implement it and so this may look different at each institution.

## The Path Forward

Even as we argue for it, we readily acknowledge the challenges of integrating CEP into CM programming. It may be difficult to initially identify a community partner and then build the trust needed for a mutually productive relationship. This task can be made more challenging if an academic institution has a troubled history with the local community. Institutions may need to work to build trust with community members and respond to evolving community needs. Institutions may also benefit, as we did, from partnering with a trusted community engagement organization.

Further, course structure and duration may limit the potential for meaningful community engagement. We recommend emphasizing practical skill development via CEP as much as classroom education and culinary didactics. If course constraints fundamentally limit the time available, we would continue to encourage an equal division of these curricular pillars, even if it comes at the expense of classroom or kitchen time. Finally, there are costs, such as compensation for faculty time, expenses for program expansion, reimbursements of community partners and student transportation, that will be incurred in extending an established CM program to include a robust community component. However, costs can be mitigated by taking advantage of existing resources (i.e., existing community institutions with this mission, volunteer forces, etc.). To justify tax-exempt status, nonprofit academic medical centers must provide community benefits; this can be emphasized to school leadership when proposing these programs. If the integration is done thoughtfully, the right community partner identified, and other institutional resources leveraged effectively, the added expenses may be well justified by the educational and community benefits described above.

Notably, a standardized definition for culinary medicine does not yet exist.^8^ As CM programming grows in popularity and becomes integrated into more academic curricula, it is important to ensure that community-engaged programming is built into CM’s definition. Early consensus criteria have recently been released about nutritional competencies for medical trainees, and multiple recommendations emphasize the importance of SDOH knowledge, cultural sensitivity, and nutritional dialogue ability.^37^ CEP in CM presents a clear opportunity for students to take what they have learned further: from the classroom, to the kitchen, to the community.

## Conclusion

The opportunity CEP provides to reinforce didactic and culinary training, foster novel forms of interprofessional training, and promote cultural and structural competence, merits its equal recognition as a key pillar of CM. As this pedagogy is integrated into CM education, it is critical that it be rigorously evaluated to establish that the practices which emerge maintain the mutually beneficial educational and community outcomes embodied by the philosophy of CEP.
